# Supplementary material for: The genus Cortinarius should not (yet) be split
Source: IMA Fungus. 2024 Aug 13;15:24. doi: 10.1186/s43008-024-00159-4 (PMC11321212; doi:10.1186/s43008-024-00159-4)
Supplement: Supplementary file 1 — Supplementary material 1 [file 43008_2024_159_MOESM1_ESM.docx]

**Supplementary Material 1**

**Taxonomic and nomenclatural superfluities**

Here we mention cases of new combinations for taxa where previous ITS barcoding (often by authors that are part of the current paper) have indicated identical barcodes of the type collections of different species. We refer to these novelties as taxonomically superfluous combinations, a term that has no standing under the rules of nomenclature. Our concept of taxonomically superfluous names refers to new combinations that are in contravention of the Preamble of the code, paragraph 12 where it is stated “*The only proper reasons for changing a name are either a more profound knowledge of the facts resulting from adequate taxonomic study or the necessity of giving up a nomenclature that is contrary to the rules*.” As no new data are available on the identity of these species, there is no proper reason for introducing many of these new combinations. This practice of taxonomically superfluous name changes also deviates from Preamble paragraph 1 that deals with the “*avoidance of the useless creation of name*s”. There are also instances of a comparable problem of potentially taxonomically superfluous names. In these cases there are no type sequences but taxonomic practice has consistently considered names to be synonyms. In Liimatainen et al. (2014) such cases are species names *sensu auct*., a practice that differs from recommendation 50D of the Code. These two categories are listed separately.

We refer to the combinations as made in Liimatainen et al. (2022), even though we do not think there is currently sufficient evidence that the genus *Cortinarius* should be split. Page numbers for species names refer to Liimatainen et al. (2022), unless indicated otherwise. The species epithets are ordered alphabetically.

1. *Phlegmacium acidophilum* (p. 139). In Liimatainen et al. (2014: 116) *Cortinarius acidophilus* is a synonym of *Cortinarius pseudonaevosus*. The combination *Phlegmacium pseudonaevosum* was subsequently made (Niskanen & Liimat. in Ind. Fung. 528: 9. 2022). However, the basionym *Cortinarius pseudonaevosus* is invalid, as it lacked a Latin description or diagnosis. The oldest available name for the species is *Cortinarius subcrassoides*, combined as *Phlegmacium subcrassoides* by Niskanen & Liimat. (in Ind. Fung. 528: 8. 2022).
2. *Calonarius barbarorum* (p. 128). In Liimatainen et al. (2014: 112) *Cortinarius barbarorum* is a synonym of *Cortinarius metarius*. The combination *Calonarius* *metarius* was made on p. 133.
3. *Phlegmacium brunneoviolaceum* (p. 141). In Liimatainen et al. (2014: 115) *Cortinarius brunneoviolaceus* is a synonym of *Cortinarius brunneolividus*. The combination *Phlegmacium brunneolividum* was made on p. 141.
4. *Phlegmacium cephalixolargum* (p. 142). In Liimatainen et al. (2014: 115) *Cortinarius cephalixolargus* is a synonym of *Cortinarius largus*. The combination *Phlegmacium largum* was made by Wünsche 1877.
5. *Phlegmacium cinctipes* (p. 142). In Liimatainen et al. (2014: 114) *Cortinarius cinctipes* is a synonym of *Cortinarius pseudocephalixus*. The combination *Phlegmacium pseudocephalixum* was made on p. 150. Note that currently the species is best known as *Cortinarius cliduchus*, but the latter name has not yet been neotypified.
6. *Phlegmacium clarum* (p. 143). In Liimatainen et al. (2014: 115) *Cortinarius clarus* is a synonym of *Cortinarius largus*. The combination *Phlegmacium largum* was made by Wünsche 1877.
7. *Phlegmacium concrescens* (p. 143). In Liimatainen et al. (2014: 115) *Cortinarius concrescens* is a synonym of *Cortinarius balteatoalbus*. The combination *Phlegmacium balteatoalbum* was made by M.M. Moser 1960.
8. *Phlegmacium congeminum* (p. 143). In Liimatainen et al. (2014: 115) *Cortinarius congeminus* is a synonym of *Cortinarius largus*. The combination *Phlegmacium largum* was made by Wünsche 1877.
9. *Thaxterogaster corrugis* (p. 159). In Liimatainen et al. (2014: 114) *Cortinarius corrugis* is a synonym of *Cortinarius turmalis*. The combination *Thaxterogaster turmalis* was made on p. 166.
10. *Thaxterogaster crenulatus* (p. 159). In Liimatainen et al. (2014: 113) *Cortinarius crenulatus* is a synonym of *Cortinarius talus*. The combination *Thaxterogaster talus* was made on p. 166.
11. *Phlegmacium cupreoviolaceum* (p. 144). In Liimatainen et al. (2014: 115) *Cortinarius cupreoviolaceus* is a synonym of *Cortinarius largus*. The combination *Phlegmacium largum* was made by Wünsche 1877.
12. *Thaxterogaster eumarginatus* (p. 160). In Liimatainen et al. (2014: 112) *Cortinarius eumarginatus* is a synonym of *Cortinarius purpurascens*. The combination *Thaxterogaster purpurascens* was made on p. 164.
13. *Calonarius frondosophilus* (p. 131). In Liimatainen et al. (2014: 112) *Cortinarius frondosophilus* is a synonym of *Cortinarius platypus*. The combination *Calonarius platypus* was made on p. 134.
14. *Thaxterogaster genuinus* (p. 160). In Liimatainen et al. (2014: 112) *Cortinarius genuinus* is a synonym of *Cortinarius collocandoides*. The combination *Thaxterogaster collocandoides* was made on p. 159.
15. *Phlegmacium josephii* (p. 145). In Liimatainen et al. (2014: 116) *Cortinarius josephii* is a synonym of *Cortinarius gracilior*. The name *Phlegmacium gracilior* was introduced by M.M. Moser 1960.
16. *Thaxterogaster largoides* (p. 161). In Liimatainen et al. (2014: 112) *Cortinarius largoides* is a synonym of *Cortinarius subpurpurascens*. The combination *Thaxterogaster subpurpurascens* was made on p 165.
17. *Phlegmacium luteovaginans* (p. 146). In Liimatainen et al. (2014: 114) *Cortinarius luteovaginans* is a synonym of *Cortinarius aurantiopallidus*. The combination *Phlegmacium aurantiopallidum* was made on p. 140.
18. *Phlegmacium misermontii* (p. 147). In Liimatainen et al. (2014: 117) *Cortinarius misermontii* is a synonym of *Cortinarius olidoamarus* var. *valentinus* (there is no type sequence available of var. *olidoamarus*). However, the combination *Phlegmacium olidoamarum* was made on p. 148.
19. *Phlegmacium muricinicolor* (p. 147). In Liimatainen et al. (2014: 115) *Cortinarius muricinicolor* is a synonym of *Cortinarius variicolor*. The combination *Phlegmacium variicolor* was made by Wünsche 1877.
20. *Thaxterogaster mutabilis* (p. 162). In Liimatainen et al. (2014: 112) *Cortinarius mutabilis* is a synonym of *Cortinarius occidentalis*. The combination *Thaxterogaster occidentalis* was made on p. 162.
21. *Thaxterogaster ochropudorinus* (p. 162). In Liimatainen et al. (2014: 113) *Cortinarius ochropudorinus* is a synonym of *Cortinarius talus*. The combination *Thaxterogaster talus* was made on p. 166.
22. *Thaxterogaster parolivascens* (p. 163). In Liimatainen et al. (2014: 114) *Cortinarius paraolivascens* is a synonym of *Cortinarius scaurus*. The combination *Thaxterogaster scaurus* was made on p. 165.
23. *Phlegmacium* *piriodolens* (p. 149). In Liimatainen et al. (2014: 115) *Cortinarus piriodolens* is a synonym of *Cortinarius variicolor.* The combination *Phlegmacium variicolor* was made by Wünsche 1877.
24. *Thaxterogaster pseudominor* (p. 164). In Liimatainen et al. (2014: 113) *Cortinarius pseudominor* is a synonym of *Cortinarius talus*. The combination *Thaxterogaster talus* was made on p. 166.
25. *Phlegmacium pseudopansa* (p. 150). In Liimatainen et al. (2014: 116) *Cortinarius pseudopansa* is a synonym of *Cortinarius varius*. The combination *Phlegmacium varium* was made by Wünsche 1877.
26. *Phlegmacium pseudopimum* (p. 150). In Liimatainen et al. (2014: 116) *Cortinarius pseudopimus* is a synonym of *Cortinarius varius*. The combination *Phlegmacium varium* was made by Wünsche 1877.
27. *Thaxterogaster pseudotalus* (p. 164). In Liimatainen et al. (2014: 113) *Cortinarius pseudotalus* is a synonym of *Cortinarius talus*. The combination *Thaxterogaster talus* was made on p. 166.
28. *Phlegmacium pseudoturmale* (p. 150). In Liimatainen et al. (2014: 115) *Cortinarius pseudoturmalis* is a synonym of *Cortinarius claricolor*. The combination *Phlegmacium claricolor* was made by A. Blytt 1905.
29. *Phlegmacium rufior* (p. 151). In Liimatainen et al. (2014: 116) *Cortinarius rufior* is a synonym of *Cortinarius varius*. The combination *Phlegmacium varium* was made by Wünsche 1877.
30. *Phlegmacium saginoides* (p. 151). In Liimatainen et al. (2014: 116) *Cortinarius saginoides* is a synonym of *Cortinarius varius*. The combination *Phlegmacium varium* was made by Wünsche 1877.
31. *Phlegmacium subaccedens* (p. 152). In Liimatainen et al. (2014: 138) *Cortinarius subaccedens* was mentioned as a very likely synonym of *Cortinarius olidoamarus* var. *valentinus*. (There is no type sequence of var. *olidoamarus*). However, the combination *Phlegmacium olidoamarum* was introduced on p. 148.
32. *Phlegmacium subamaricatum* (p. 152). In Liimatainen et al. (2014: 116) *Cortinarius subamaricatus* is a synonym of *Cortinarius tirolianus*. The combination *Phlegmacium tirolianum* was made on p. 154.
33. *Phlegmacium subcyanites* (p. 152). In Liimatainen et al. (2014: 114) *Cortinarius subcyanites* is a synonym of *Cortinarius cyanites*. The combination *Phlegmacium cyanites* was made by M.M. Moser 1960.
34. *Phlegmacium subdecoloratum* (p. 153). In Liimatainen et al. (2014: 114) *Cortinarius subdecoloratus* is a synonym of *C. ochraceobrunneus*. The *combination Phlegmacium ochraceobrunneum* was made on p. 148.
35. *Phlegmacium subfuligineum* (p. 153). In Liimatainen et al. (2014: 116) *Cortinarius subfuligineus* is a synonym of *Cortinarius subrugulosus*. The combination *Phlegmacium subrugulosum* was made on p. 153.
36. *Thaxterogaster subinops* (p. 165). In Liimatainen et al. (2014: 112) *Cortinarius subinops* is a synonym of *Cortinarius subpurpurascens*. The combination *Thaxterogaster subpurpurascens* was made on p. 165.
37. *Phlegmacium subvariiforme* (p. 154). In Liimatainen et al. (2014: 116) *Cortinarius subvariiformis* is a synonym of *Cortinarius luteocingulatus*. The combination *Phlegmacium luteocingulatum* was made on p. 146.
38. *Thaxterogaster thalliopurpurascens* (p. 166). In Liimatainen et al. (2014: 114) *Cortinarius thalliopurpurascens* is a synonym of *C. herpeticus*. The combination *Thaxterogaster herpeticus* was made on p. 118.
39. *Phlegmacium vacciniophilum* (p. 155). In Liimatainen et al. (2014: 116) *Cortinarius vacciniophilus* is a synonym of *Cortinarius pseudonaevosus*. The combination *Phlegmacium pseudonaevosum* was subsequently made (Niskanen & Liimat. in Ind. Fung. 528: 9. 2022). However, the basionym *Cortinarius pseudonaevosus* is invalid, as it lacked a Latin description or diagnosis. The oldest available name for the species is *Cortinarius subcrassoides*, combined as *Phlegmacium subcrassoides* by Niskanen & Liimat. (in Ind. Fung. 528: 8. 2022).

The second category pertains to names that are likely taxonomically superfluous. These cases involve species names that have not been typified but have been and still are in current use, and where the taxonomic interpretation does not seem to be in doubt. Such cases have been indicated in Liimatainen et al. (2014) as *sensu auct.* This terminology is potentially misleading, as it involves a different interpretation of that concept than specified in the rules of nomenclature (Recommendation 50D, where misidentifications are referred to as misapplications, which should be indicated by “auct., non [followed by the name of the original author]). In some rare cases Liimatainen et al. (2022) did accept the names in its current interpretation and made new combinations (e.g., *Thaxterogaster vespertinus* on p. 166, where the possible new combination based on *Cortinarius variipes* has not been made; *Calonarius sulfurinus* on p. 136, for which no synonyms or misapplications were reported). In other, more frequent, cases they combined both the name in current use (s. auct.) and very likely younger synonyms. These cases are:

1. *Calonarius alnobetulae* (p. 127). In Liimatainen et al. (2014: 113) *Cortinarius alnobetulae* is considered the same species as *Cortinarius moseri* s. auct. The combination *Calonarius alnobetulae* was made on p. 133.
2. *Calonarius calojanthinus* (p. 129). In Liimatainen et al. (2014: 112) *Cortinarius calojanthinus* is considered the same species as *Cortinarius corrosus* s. auct. The combination *Calonarius corrosus* was made on p. 130.
3. *Calonarius elotoides* (p. 130). In Liimatainen et al. (2014: 112) *Cortinarius elotoides* is considered the same species as *Cortinarius pseudoglaucopus* s. auct. The combination *Calonarius pseudoglaucopus* was made on p. 135.
4. *Calonarius evosmus* (p. 130). In Liimatainen et al. (2014: 112) *Cortinarius evosmus* is considered the same species as *Cortinarius osmophorus* s. auct. The combination *Calonarius osmophorus* was made on p. 135.
5. *Phlegmacium flavescentipes* (p. 144). In Liimatainen et al. (2014: 116) *Cortinarius flavescentipes* is considered the same species as *Cortinarius balteatocumatilis* s. auct. The combination *Phlegmacium balteatocumatile* was made on p. 141.
6. *Phlegmacium gentianeum* (p. 145). In Liimatainen et al. (2014: 117) *Cortinarius gentianeus* is considered the same species as *Cortinarius caesiostramineus* s. auct. The combination *Phlegmacium caesiostramineum* was made by M.M. Moser 1960.
7. *Calonarius juxtadibaphus* (p. 132). In Liimatainen et al. (2014: 113) *Cortinarius juxtadibaphus* is considered the same species as *Cortinarius dibaphus* s. auct. The combination *Calonarius dibaphus* was made on p. 130.
8. *Phlegmacium latoclaricolor* (p. 146). In Liimatainen et al. (2014: 115) *Cortinarius latoclaricolor* is considered the same species as *Cortinarius durus* s. auct. The combination *Phlegmacium durum* (as *P. durus*) was made on p. 144.
9. *Phlegmacium leonicolor* (p. 146). In Liimatainen et al. (2014: 116) *Cortinarius leonicolor* is considered the same species as *C. anserinus* s. auct. and *C. amoenolens* s. auct. The name *Phlegmacium anserinum* Velen. dates from 1920, whereas the new combination *Phlegmacium amoenolens* was made on p. 139. *Cortinarius gratus* is equally considered a synonym in Liimatainen et al. (2014: 116), but the combination *Phlegmacium gratum* was additionally made by Niskanen & Liimat. (in Ind. Fung. 528: 8. 2022).
10. *Thaxterogaster mendax* (p. 162). In Liimatainen et al. (2014: 112) *Cortinarius mendax* is considered the same species as *Cortinarius subporphyropus* s. auct. The combination *Thaxterogaster subporphyropus* was made on p. 165.
11. *Phlegmacium neotriumphans* (p. 147). In Liimatainen et al. (2014: 114) *Cortinarius triumphans* is considered the same species as *C. saginus* s. auct. The combination *Phlegmacium saginum* was made on p. 114. However, it had been previously made by Ricken 1912.
12. *Phlegmacium ophiopus* (p. 148). In Liimatainen et al. (2014: 116) *Cortinarius ophiopus* is considered the same species as *Cortinarius triumphans* s. auct. The combination *Phlegmacium triumphans* was made by A. Blytt 1905.
13. *Calonarius rapaceoides* (p. 135). In Liimatainen et al. (2014: 112) *Cortinarius rapaceoides* is considered the same species as *Cortinarius caroviolaceus* s. auct. The combination *Calonarius caroviolaceus* was made on p. 129.
14. *Phlegmacium scaurocaninus* (p. 151). See below. In Liimatainen et al. (2014: 117) *Cortinarius scaurocaninus* is considered the same species as *C. magicus* s. auct.
15. *Calonarius subpurpureophyllus* (p. 136). In Liimatainen et al. (2014: 113) *Cortinarius subpurpureophyllus* is considered the same species as *C. napus* s. auct. The combination *Calonarius napus* was made on p. 133.
16. *Phlegmacium triumphale* (p. 154). In Liimatainen et al. (2014: 115) *Cortinarius triumphalis* is considered the same species as *C. vulpinus* s. auct. The combination *Phlegmacium vulpinum* was made on p. 155.
17. *Phlegmacium veneris* (p. 155). In Liimatainen et al. (2014: 116) *Cortinarius veneris* is considered the same species as *Cortinarius balteatocumatilis* s. auct. The combination *Phlegmacium balteatocumatile* was made on p. 141.

Finally, we list two cases of evident generic misclassifications.

1. The combination *Calonarius coniferarum* was introduced on p. 130. Its basionym, *Phlegmacium multiforme* var. *coniferarum*, is generally considered closely related to or a synonym of *C. multiformis*, as species classified in *Thaxterogaster*. However, it is plausible that they intended to recombine *Phegmacium callochroum* var. *coniferarum* in *Calonarius* on species level. But that taxon is considered to be a synonym of *Cortinarius piceae*, which they also recombined as *Calonarius piceae* on p. 134.
2. The combination *Thaxterogaster magicus* was introduced on p. 161. In a subsequent ‘correction’ the combination *Phlegmacium magicum* was made by Niskanen & Liimat. (in Ind. Fung. 536: 4. 2023). However, in Liimatainen et al. (2014: 117) *Cortinarius scaurocaninus* is considered the same species as *C. magicus* s. auct. and the combination *Phlegmacium scaurocaninum* was made on p. 151. However, whereas *Cortinarius magicus* replaced the illegitimate *Cortinarius subglaucopus* Eichhorn 1953 (non *C. subglaucopus* Murrill 1939), the new name *Phlegmacium subglaucopus* M.M. Moser (Gattung Phlegmacium: 199. 1960) is legitimate under Art. 58 and the oldest name for this species.
