# Supplementary figures and images for: The genus Cortinarius should not (yet) be split
Source: IMA Fungus. 2024 Aug 13;15:24. doi: 10.1186/s43008-024-00159-4 (PMC11321212; doi:10.1186/s43008-024-00159-4)

Gene  
Present

Gene  
Absent

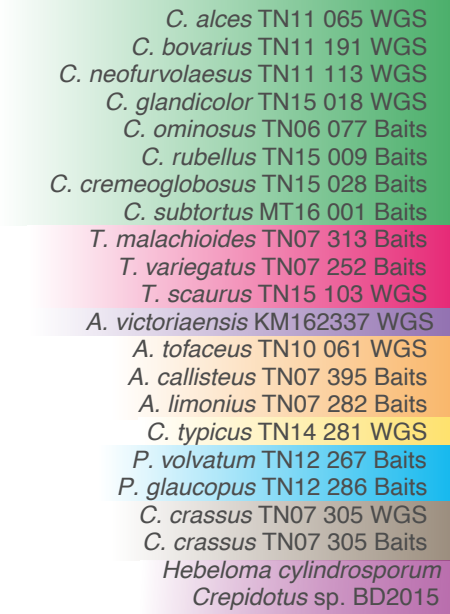

Supplement: Supplementary file 2 — Supplementary material 2 [file 43008_2024_159_MOESM2_ESM.pdf]

A

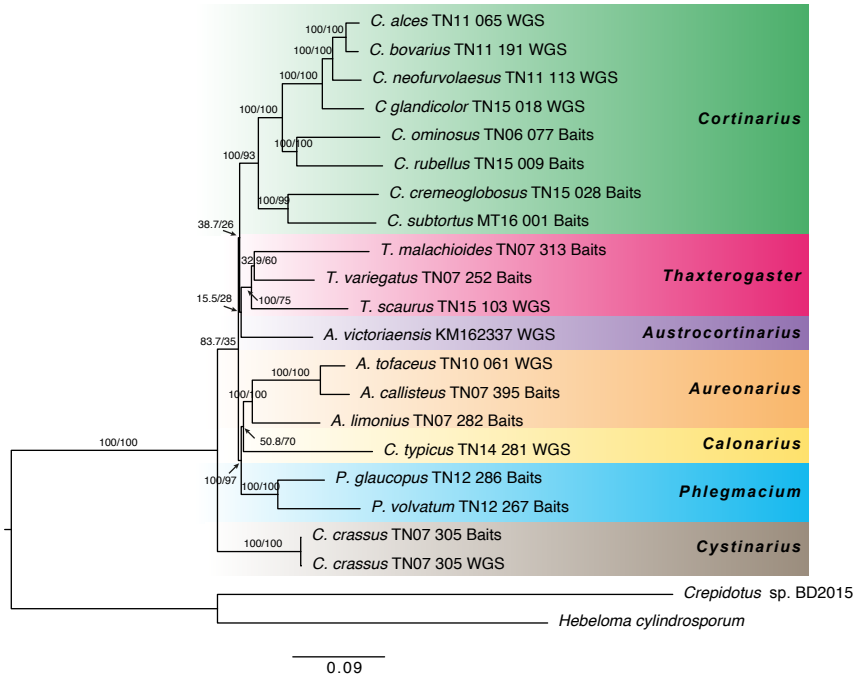

B

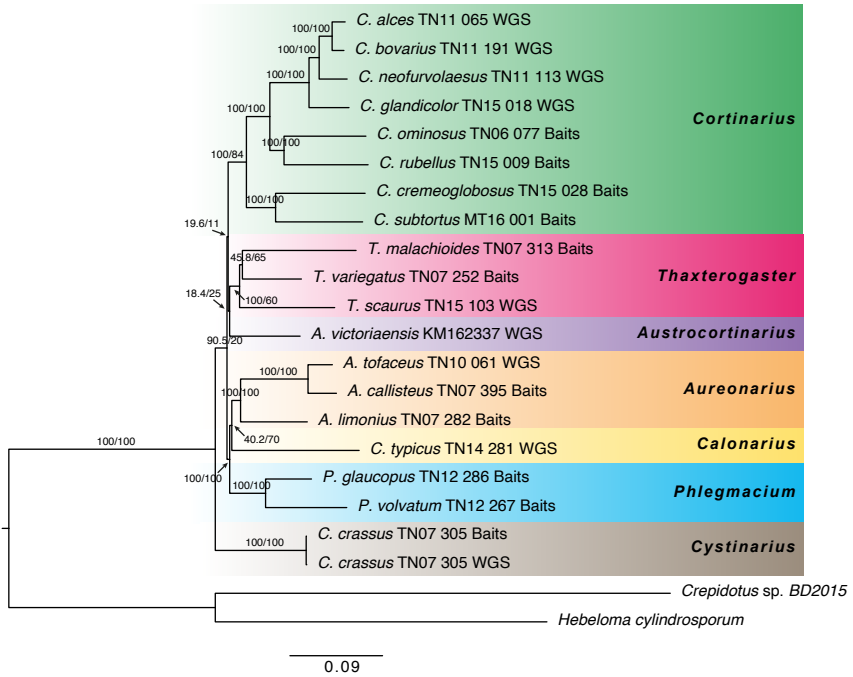

C

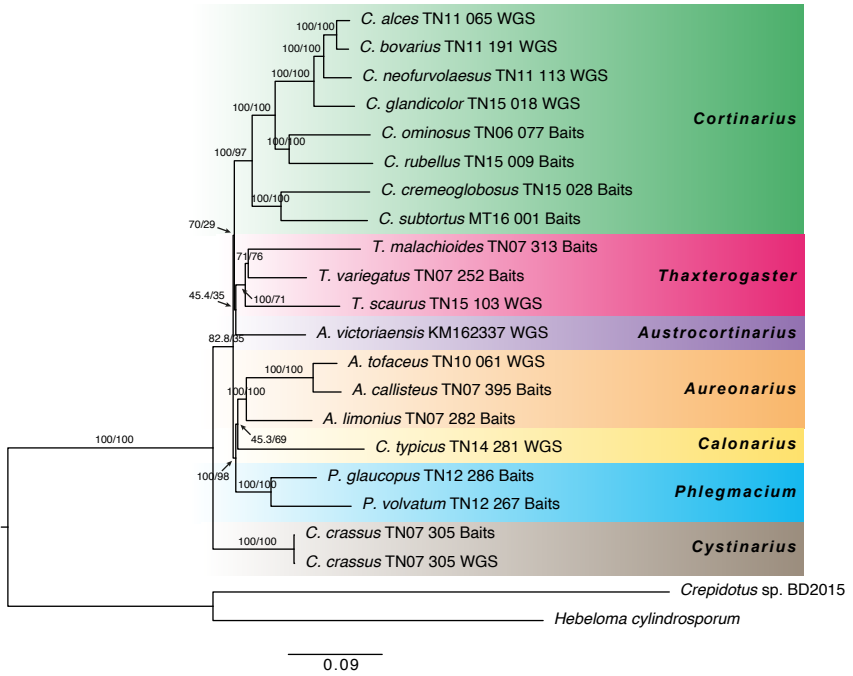

Supplement: Supplementary file 3 — Supplementary material 3 [file 43008_2024_159_MOESM3_ESM.pdf]

A

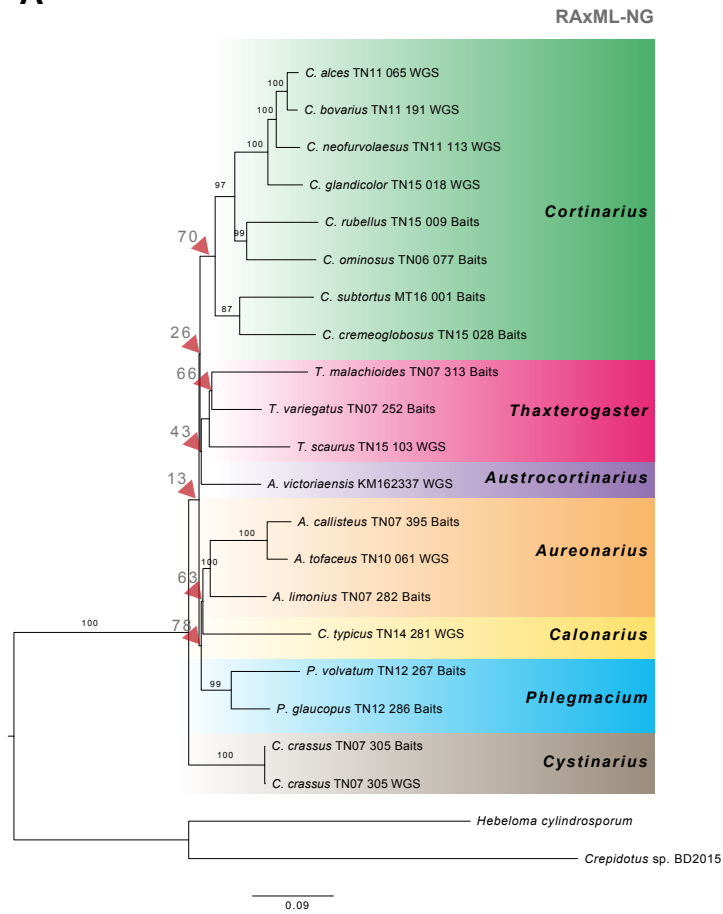

B

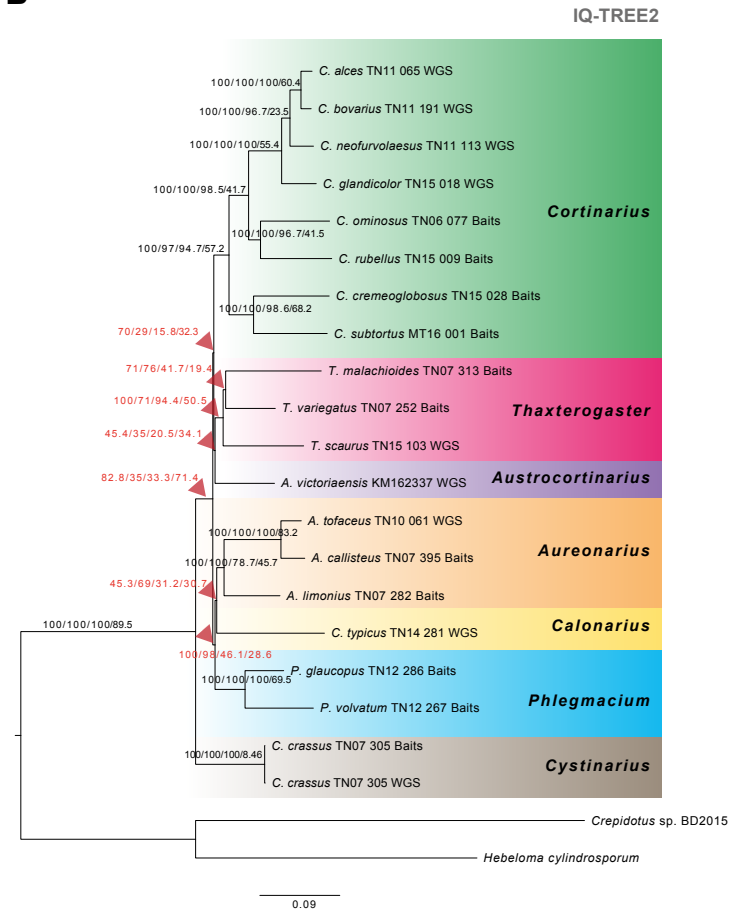

C

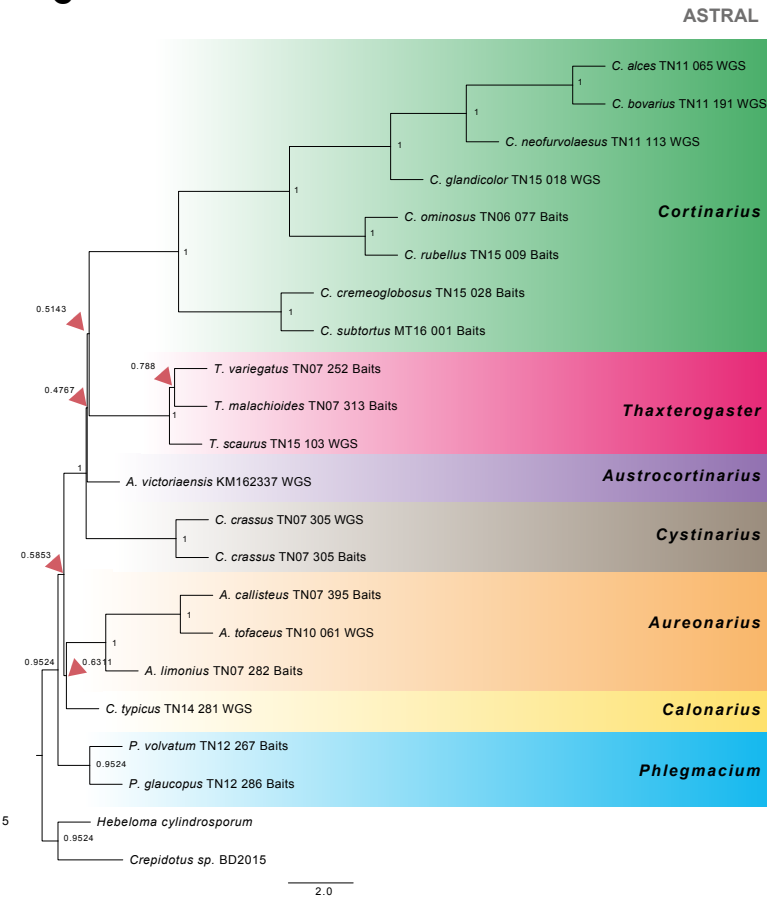

Supplement: Supplementary file 4 — Supplementary material 4 [file 43008_2024_159_MOESM4_ESM.pdf]
